# Mandatory role of endoplasmic reticulum and its pentose phosphate shunt in the myocardial defense mechanisms against the redox stress induced by anthracyclines

**DOI:** 10.1007/s11010-023-04903-z

**Published:** 2023-12-12

**Authors:** Gianmario Sambuceti, Vanessa Cossu, Francesca Vitale, Eva Bianconi, Sonia Carta, Consuelo Venturi, Sabrina Chiesa, Francesco Lanfranchi, Laura Emionite, Sebastiano Carlone, Luca Sofia, Francesca D’Amico, Tania Di Raimondo, Silvia Chiola, Anna Maria Orengo, Silvia Morbelli, Pietro Ameri, Matteo Bauckneht, Cecilia Marini

**Affiliations:** 1 IRCCS Ospedale Policlinico San Martino, 16132 Genoa, Italy; 2 Department of Health Sciences, University of Genoa, 16132 Genoa, Italy; 3 Department of Experimental Medicine, Human Anatomy Section, University of Genoa, 16132 Genoa, Italy; 4 Department of Internal Medicine, University of Genoa, 16132 Genoa, Italy; 5 Institute of Molecular Bioimaging and Physiology (IBFM), National Research Council (CNR), 20054 Milan, Italy

**Keywords:** Myocardial metabolism, Anthracycline toxicity, Cancer, Pentose-phosphate pathway, PET imaging

## Abstract

Anthracyclines’ cardiotoxicity involves an accelerated generation of reactive oxygen species. This oxidative damage has been found to accelerate the expression of hexose-6P-dehydrogenase (H6PD), that channels glucose-6-phosphate (G6P) through the pentose phosphate pathway (PPP) confined within the endoplasmic/sarcoplasmic reticulum (SR). To verify the role of SR-PPP in the defense mechanisms activated by doxorubicin (DXR) in cardiomyocytes, we tested the effect of this drug in H6PD knockout mice (H6PD^−/−^). Twenty-eight wildtype (WT) and 32 H6PD^−/−^ mice were divided into four groups to be treated with intraperitoneal administration of saline (untreated) or DXR (8 mg/Kg once a week for 3 weeks). One week thereafter, survivors underwent imaging of ^18^F-deoxyglucose (FDG) uptake and were sacrificed to evaluate the levels of H6PD, glucose-6P-dehydrogenase (G6PD), G6P transporter (G6PT), and malondialdehyde. The mRNA levels of SR Ca^2+^-ATPase 2 (*Serca2*) and ryanodine receptors 2 (*RyR2*) were evaluated and complemented with Hematoxylin/Eosin staining and transmission electron microscopy. During the treatment period, 1/14 DXR-WT and 12/18 DXR-H6PD^−/−^ died. At microPET, DXR-H6PD^−/−^ survivors displayed an increase in left ventricular size (*p* < 0.001) coupled with a decreased urinary output, suggesting a severe hemodynamic impairment. At ex vivo analysis, H6PD^−/−^ condition was associated with an oxidative damage independent of treatment type. DXR increased H6PD expression only in WT mice, while G6PT abundance increased in both groups, mismatching a generalized decrease of G6PD levels. Switching-off SR-PPP impaired reticular accumulation of Ca^2+^ decelerating *Serca2* expression and upregulating *RyR2* mRNA level. It thus altered mitochondrial ultrastructure eventually resulting in a cardiomyocyte loss. The recognized vulnerability of SR to the anthracycline oxidative damage is counterbalanced by an acceleration of G6P flux through a PPP confined within the reticular lumen. The interplay of SR-PPP with the intracellular Ca^2+^ exchanges regulators in cardiomyocytes configure the reticular PPP as a potential new target for strategies aimed to decrease anthracycline toxicity.

## Introduction

The therapeutic effectiveness of anthracyclines is flawed by a high incidence of life-threatening cardiac toxicity that can occur either acutely or up to years after administration [1]. Although the underlying mechanisms have not been fully defined, a wide literature identified the oxidative damage as a main mechanism of anthracyclines’ cardiotoxicity due to the autoxidation property of drug semiquinone moiety amplified by the high oxygen tension of the myocardium [2]. According to this paradigm, the enhanced generation of reactive oxygen species (ROS) causes a pathological leakage of Ca^2+^ ions from the endoplasmic/sarcoplasmic reticulum (ER/SR), eventually triggering a programmed cell death [3, 4].

Cardiomyocyte antioxidant responses largely rely on glutathione- and thioredoxin-dependent pathways and thus on the NADPH reductive power, primarily provided by an accelerated glucose-6-phosphate (G6P) flux through the pentose phosphate pathway (PPP). Nevertheless, this consideration has been challenged by the evidence that the deficiency of G6P-dehydrogenase (G6PD) is not associated with an increased risk of cardiac complications, at least in two large cohorts of pediatric patients with acute lymphoblastic leukemia submitted to anthracycline-based chemotherapy [5].

This clinical observation seemingly indicates a marginal role of PPP in the prevention of anthracycline cardiotoxicity. Nevertheless, mammalian cells also run a further PPP, confined within the ER/SR, and largely autonomous from its well-recognized cytosolic counterpart [6, 7]. We previously showed that the increased ROS content and oxidative damage induced by doxorubicin (DXR) accelerate the cardiomyocyte expression of hexose-6P-dehydrogenase (H6PD) [8], i.e., the enzyme triggering an autonomous PPP within the ER/SR.

Actually, the ER/SR is the principal regulator of Ca^2+^ storage and release as thus represents a major determinant of cardiomyocyte systolic and diastolic function [9]. However, the ER/SR also plays a pivotal role in regulating Ca^2+^ transfer into mitochondria opening the permeability transition pore as to the release of cytochrome *c* from the mitochondrial matrix [10]. The consequent inhibition of complex III of the electron transport chain eventually increases the ROS generation [11], triggering a vicious cycle that eventually disrupts cardiomyocyte homeostasis up to induction of apoptosis [12]. These considerations agree with the acknowledged link between ER/SR vulnerability to anthracycline toxicity and the high incidence of myocardial dysfunction in patients submitted to this treatment [3, 4]. In agreement with this concept, it is not surprising that the ER/SR is enriched with a local control of redox homeostasis, most likely favored by the presence of a confined PPP, triggered by H6PD.

These considerations agree with the high predictive power of myocardial uptake of ^18^F-Fluoro-deoxyglucose (FDG) for the subsequent occurrence of cardiac disorders in patients treated with DXR because of Hodgkin lymphoma [13]. Indeed, although the retention of this tracer is classically considered to represent an index of overall glucose consumption, recent evidence documented that FDG uptake is strictly dependent upon the catalytic function of H6PD [14]. This “omnivore” enzyme can process a variety of free and phosphorylated hexoses, including FDG-6P as to prevent the radioactivity release related to its dephosphorylation catalyzed by the ubiquitous G6P-phosphatase *β*, at least in cancer [15], neurons [16] and skeletal muscle [17].

We thus aimed to verify whether and to what degree the ER/SR-PPP contributes to the myocardial response to anthracycline toxicity in experimental models of H6PD gene deletion.

## Methods

### Animal models

All experiments were performed in accordance with the guidelines established by the European Community Council (Directive 114 2010/63/EU of September 22, 2010). They were reviewed and approved by the Licensing and Animal Welfare Body of the Italian Ministry of Health (Authorization n° 99/2021-PR, in compliance with the Italian law, D.Lgs. 26/2014).

The study included 28 wildtype C57BL/6 J mice (WT) as a control group and 32 littermates, global constitutive knock-out (KO) for the H6PD encoding gene (H6PD^−/−^) obtained by inbreeding of animals kindly provided by dr. Lavery, University of Birmingham, UK [18]. All mice were housed in the animal facility of the IRCCS—Ospedale Policlinico San Martino, on a 12 h/12 h light–dark cycle with free access to standard rodent chow and water. The two colony precursors heterozygote mice were intercrossed to generate WT, heterozygote and H6PD^−/−^ mice. Genotypes were routinely monitored using Polimerase Chain reaction (PCR) with DNA extracted from tail biopsy, according to the methodology described by Lavery et al. [18].

The 28 WT mice were divided into two groups to be treated with either intraperitoneal administration of DXR (*n* = 14), or an equivalent volume of saline (*n* = 14). The H6PD^−/−^ group included 32 mice, since the high mortality rate caused by DXR brought us to include 18 further mice as opposed to the 14 ones treated with saline. Saline or DXR were administered were performed once a week for 3 weeks (day 1, 7 and 14). Drug dose was 8 mg/Kg body weight for a cumulative dose of 24 mg/Kg [19]. All in vivo and ex vivo analysis were performed 1 week after the last treatment and were thus restricted to survivors.

### Isolation of liver mitochondrial and microsomal fractions

To obtain data comparable with previous literature [20], the analysis of H6PD localization implied the isolation of mitochondria and microsome fractions of freshly harvested hepatocytes. All manipulations were performed at 4 °C. Samples were homogenized using homogenization buffer (0.25 M sucrose, 0.15 M KCl, 1 Mm EDTA, 10 Mm Tris–HCl pH 7.4, 1:8 weight/volume) and then centrifuged at 800 × g for 10 min in an Eppendorf Centrifuge, to precipitate nuclei and cellular debris. The collected supernatant was further centrifuged for 15 min at 12,000 × g. Thereafter, the resulting supernatant was further centrifuged for 40 min at 100,000 × g in a ProteomeLab XL-A/XL-I Beckman centrifuge (Beckman Coulter, Brea, CA, USA), to obtain the microsomal fraction [21]. The pellet obtained after the 12,000 g centrifugation, containing mitochondria, was resuspended in a solution buffer (0.225 M sucrose, 0.075 M mannitol, 10 mM Tris–HCl pH 7.4, 1 mM EDTA), centrifuged for 10 min at 1,000 × g and washed by centrifugation at 12,000 g for 15 min [22].

### Experimental microPET imaging

At day #21, and thus 1 week after the end of treatment, all surviving animals were submitted to microPET scanning (Albira, Bruker, USA), according to our standard protocol [8, 15–17]. Briefly, WT and H6PD^−/−^ animals underwent 6 h fasting, with free access to water, prior to anesthetization by intraperitoneal administration of ketamine 100 mg/Kg + xylazine 10 mg/Kg. Thereafter, mice were weighted, and serum glucose level was assayed soon before the administration of 3–4 MBq of FDG through a tail vein. A dynamic acquisition was performed and binned in five frames, each lasting 10 min.

PET data were reconstructed using a maximal likelihood expectation maximization method (MLEM). The last “steady state” frame was analyzed using a dedicated software (PMOD, Bruker). An experienced observer, unaware of the experimental condition, drew a volume of interest (VOIs) to estimate average and maximal heart radioactivity that was expressed as standardized uptake value (SUV), conventionally defined as:

$${\text{SUV}} = \frac{{{\text{Local}} \;{\text{radioactivity}}\;{\text{ concentration}} \left( {\frac{{{\text{KBq}}}}{{{\text{mL}}}}} \right) \times {\text{Body}} {\text{weight}} \left( {{\text{Kg}}} \right)}}{{{\text{Administered }}\;{\text{dose}} \left( {{\text{MBq}}} \right)}}.$$

Finally, the dynamic acquisition was exploited to verify the absence of any urine loss during scan duration and the urinary tracer excretion was estimated according to the following equation:

$${\text{Urinary }}\;{\text{FDG }}\;{\text{excretion}} = \frac{{{\text{Total}} \;{\text{bladder}}\;{\text{ radioactivity }}\;{\text{content}} \left( {{\text{KBq}}} \right)}}{{{\text{Total }}\;{\text{body}}\;{\text{ radioactivity}}\;{\text{ content}} \left( {{\text{KBq}}} \right)}} \left( \% \right).$$

The day after the in vivo experiments, all animals were sacrificed and tissues were harvested, divided, and properly preserved for ex vivo analysis.

### Ex vivo analysis

Ex vivo analysis of malondialdehyde (MDA), protein expression and mRNA levels were performed in ≥ 3 samples of hearts harvested from survivors to both treatment and PET. All harvested hearts were divided and stored at −80 °C. Frozen samples were homogenized using the RIPA buffer (10 mM Tris–HCl, pH 8.0 1 mM EDTA, 0.5 mM EGTA, 1% Triton X-100, 0.1% Sodium Deoxycholate, 0.1% SDS, 140 mM NaCl) and sonicated twice for 10 s (s) in ice, with a break of 30 s. Enzymatic assays were performed in frozen homogenates or subcellular fractions, using a UV/visible spectrophotometer (Ultraspec 2000, Pharmacia Biotech, Erie, PA, USA). In mitochondrial and microsomal fractions isolated from liver, H6PD and G6PD activities were assayed following reduction of NADP, at 340 nm. The following assays solutions were used: H6PD: Tris–HCl pH 7.4, glucose 10 mM, NADP 0.5 mM; G6PD: Tris–HCl 7.4, G6P 10 mM, and NADP 0.5 mM.

MDA levels were evaluated by the thiobarbituric acid reactive substances (TBARS) assay, with minor modifications [8, 17]. Total antioxidant capacity was evaluated following the instructions of the manufacturer of a dedicated kit (Abcam; Cat #ab65329, Cambridge, UK), expressed as Trolox equivalent antioxidant capacity content. All assays were normalized for total protein concentrations tested using Bradford analysis.

Western blot (WB) experiments were performed according to the standard procedure. Proteins were extracted using RIPA buffer supplemented with protease inhibitor cocktail. Denaturing electrophoresis (SDS-PAGE) was performed using Mini PROTEAN TGX Precast gels (Bio-Rad, Hercules, CA, USA) and 15 μg of total protein were loaded for each sample. Running buffer contained 25 mM Tris-Base (pH 8.0), 0.192 mM glycine and 0.1% SDS. Electrophoretically separated samples were transferred onto PVDF membranes by electroblotting, at 400 mA for 1:30 h (h) in Tris–glycine buffer (25 mM Tris and 0.192 mM glycine) plus 20% (v/v) methanol, at 4 °C. After blocking unspecific protein biding-sites with 5% non-fat milk in Tris Buffer Saline + 0.10% Tween20 (TBST), PVDF membranes were incubated with the following antibodies (Abs): anti-H6PD (#ab170895, Abcam), anti-G6PD (#ab124738, Abcam), anti-G6P transporter (G6PT, #ab124738, Abcam), anti-calnexin (Clnx, #2679, Cell Signaling Technology, Danvers, Massachusetts, USA) anti-mitofusin 2 (MFN2, #PA5-72,811, ThermoFisher Scientific, Waltham, MA, USA), and anti-alpha-Tubulin (T6074, Sigma, St. Louis, MO, USA). Appropriate secondary horseradish peroxidase-conjugated Abs, diluted 1:10,000, were incubated for 1 h. Bands were detected and analyzed for optical density using an enhanced chemiluminescence substrate (ECL, Bio-Rad), a chemiluminescence system (Alliance Mini HD, UVITEC, Cambridge, UK), and NineAlliance software (UVITEC). All bands of interest were normalized with alpha-Tubulin levels detected on the same membrane.

For Real-Time Polymerase Chain Reaction analysis (RT-PCR), total RNA was purified using a Quick RNA Miniprep Plus kit (Zymo Research, Irvine, CA, USA) according to the manufacturer’s instructions and reverse transcribed using the SensiFAST cDNA Synthesis KIT (Meridian Life Science, Inc., Memphis, TE, USA). RT-PCR was performed using SensiFAST SYBR (Bioline). Primers were designed using PRIMER 3 software and purchased from TIB MOLBIOL (Genoa, Italy). The following gene levels were analyzed: ER/SR Ca^2+^-ATPase 2 (Atp2a2, alias *Serca2*), ryanodine receptors 2 (RyR2). Target gene levels were normalized to that of TATA-box binding protein (TBP) mRNA. The Q-Gene program was used for analyzing gene expressions [23].

### Histological and ultrastructural analysis

Soon after sacrifice, three hearts for each group were embedded in optimal cutting temperature polymer (OCT). Serial eight-micrometer-thick cryostat sections were then obtained and stained with Hematoxylin/Eosin staining. Briefly, heart sections were fixed in neutral buffered formalin 10% (Meccanica G.M. S.r.l., Loreto, AN, Italy) for 15 min at room temperature and then incubated for 40 s in Harris’ Hematoxylin (Bio-Optica Milano S.p.a., Milano, Italy), rinsed in tap water and lastly incubated for 20 s in Eosin Y (SIGMA; 0.5% in deyonized water and supplemented with a 0.2% of acetic acid). Slides were then dehydrated, cleared, and mounted with Bio Mount HM (Bio-Optica). Images were obtained both using Leica DM 2000 LED microscope (Leica Microsystem CMS GmbH, Wetzlar, Germany) in bright field at 20X of magnification, ToupCam Camera E3ISPM and ToupView v. × 64 software (ToupTek Photonics, Zhejiang, China) and with the slide scanner Aperio AT2 (Leica Biosystem) with its associated software Aperio ImageScope. Nuclear density was measured by an observer unaware of the of the experimental model. At least 20 fields (100 × 100 µm) were analyzed using the dedicated routine of NIH ImageJ software (Fiji 1.0) and expressed as number of nuclei /mm^2^.

For ultrastructural analysis, three hearts per group were dissected and fixed in 0.025 M cacodylate buffer containing 2.5% glutaraldehyde (Electron Microscopy Science, Hatfield, PA, USA), for 4 h at room temperature. Samples were postfixed in osmium tetroxide 2% overnight. Samples were next dehydrated through a graded ethanol series and acetone and embedded in epoxy resin (Poly-Bed; Polysciences, Inc., Warrington, PA, USA) overnight at 42 °C and 2 days at 60 °C. Ultrathin Sects. (50 nm) were counterstained with uranyless and lead citrate. Transmission electron microscopy (TEM) images were thus obtained using the microscope HT7880 (Hitachi, Japan).

On obtained images, mitochondria were manually outlined by two investigators unaware of the experimental group to define visible mitochondrial area in µm^2^. Further, *cristae* abundance was evaluated according to the criterion proposed by Lam et al. [24], modified to disregard the qualitative evaluation of *cristae* structure focusing on their abundance. The used score was thus: 0 no sharply defined *cristae*; 1: ≥ 75% of the mitochondrial area without *cristae*, 3: < 25% of mitochondrial area without *cristae*.

### Statistical analysis

Based on our previously published data [8, 9], we hypothesized that DXR would have increased myocardial-SUV by about 45 ± 18% in WT mice. By contrast, based on the effect of H6PD silencing in cancer cells, we hypothesized that H6PD silencing would have decreased this response by 33 ± 10%. Using a free on-line tool (http://www.openepi.com/SampleSize/SSMean.htm) sample size was estimated at ≥ 12 mice per group. Nevertheless, the high death rate following DXR, brought us to increase the sample size of the H6PD^−/−^ group, to obtain an adequate number of analyzable specimens.

Data are presented as mean ± standard deviation (SD). Multiple comparisons among the studied groups were performed by two-way analysis of variance (ANOVA) with Tukey’s multiple comparisons test. Synergism between H6PD gene expression and DXR treatment was tested using factorial experiment design with the interaction factor estimated using the univariate analysis of the general linear regression model as described by Slinker [25]. Statistical significance was considered for *p* values < 0.05. Statistical analyses were performed using SPSS software Advanced Models 15.0 (Chicago, Illinois).

## Results

### Reticular localization of H6PD and its impact on mouse phenotype

**Fig. 1:**
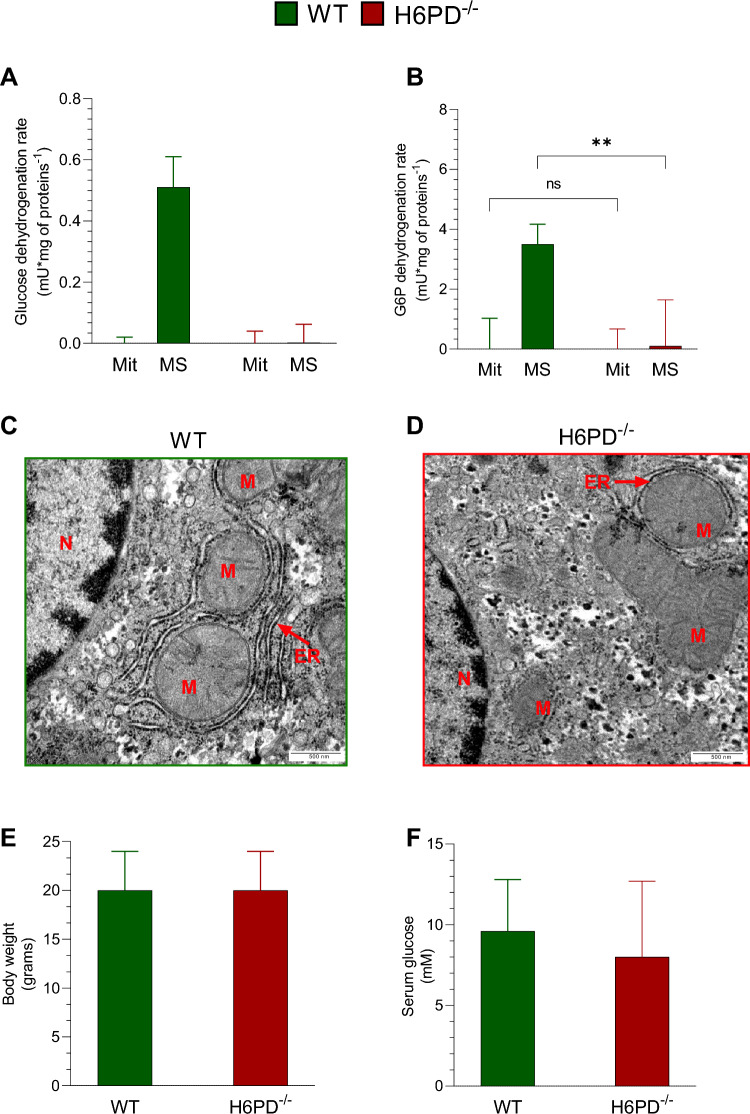
H6PD intracellular localization and its role in ER-mitochondria organization. **A** H6PD activity, expressed as glucose dehydrogenation rate, and **B** G6PD activity, expressed as the G6P dehydrogenation rate, of mitochondria (Mit) and microsomal fraction (MS) harvested from hepatocytes of wild-type (WT, green columns) and H6PD KO (H6PD^−/−^, red columns) mice before the start of doxorubicin treatment. Representative electron microscopy images of liver harvested from WT (**C**) and H6PD (**D**) mice, showing nucleus (N) and the ultrastructure of mitochondria (M) and endoplasmic reticulum (ER). **E** Body weight and **F** serum glucose concentration of WT (green columns) and H6PD^−/−^ (red columns) mice before the start of doxorubicin treatment. Data are expressed as mean ± SD. ** = *p* < 0.01

As a preliminary step, the intracellular localization of H6PD and the response of G6PD catalytic function to the shut-down of glucose flux through the ER/SR-PPP was analyzed in the hepatocytes of three untreated mice of both WT and global H6PD^−/−^ groups. H6PD catalytic function, expressed as the NADP^+^-dependent glucose dehydrogenation capability, was virtually absent in both cell fractions of KO mice. By contrast, it was well and selectively represented in the microsomal fraction of WT ones (Fig. 1A). Similarly, G6PD activity, estimated by the rate of NADP-dependent G6P dehydrogenation rate, showed the expected behavior: it was comparable in hepatocytes’ homogenates (data not shown) and absent in the mitochondria of both groups (Fig. 1B). Intriguingly, this reaction was also catalyzed by the microsomal fractions of WT mice, apparently suggesting the presence of G6PD activity bound the outer ER membranes.

Analysis of TEM images showed that H6PD gene deletion was associated with a profound alteration of ER/mitochondria networking. Indeed, the strict connection between the mitochondria and the ER observed in WT models was markedly altered and virtually absent in KO mice (Fig. 1C, D).

Nevertheless, constitutive H6PD gene deletion did not affect mouse growth with body weight being superimposable in WT and H6PD^−/−^ mice (Fig. 1E) at the study start. Likewise, enzyme deficiency did not affect fasting serum glucose level that was similar in the two groups (Fig. 1F).

### H6PD deficiency and DXR-related toxicity

**Fig. 2:**
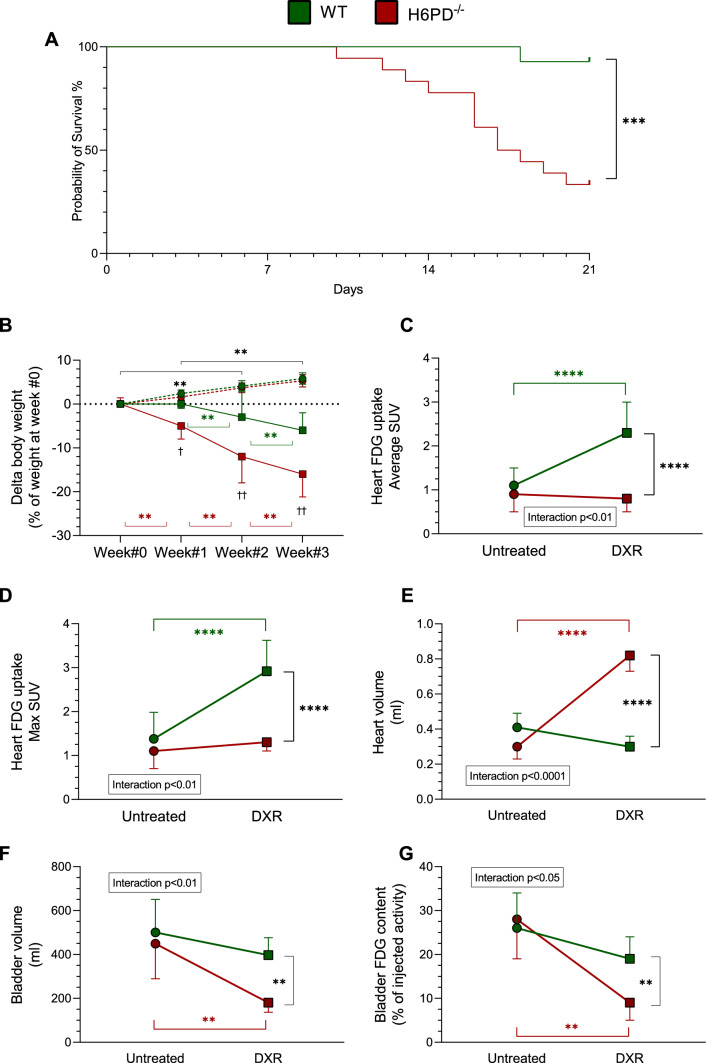
DXR treatment effect on survival, body weight and FDG uptake of H6PD knock-out mice. **A** Kaplan-Meyer curves for the survival time during doxorubicin treatment in wild-type (WT, green line) and H6PD knock-out (H6PD^−/−^, red line) mice. **B** Weight changes over time, expressed as % of weight at start of treatment (week #0), in WT (green line) and H6PD^−/−^ (red line) mice of untreated (circle) and doxorubicin treated (square) groups. **C** Myocardial FDG average Standardized Uptake Value (SUV), **D** myocardial maximal SUV and **E** heart volume of WT (green) and H6PD^−/−^ (red) mice of untreated and doxorubicin treated (DXR) groups evaluated 1 week after the last injection of doxorubicin. **F** Bladder volume and FDG content in untreated and DXR groups of WT (green) and H6PD^−/−^ (red) mice. Data are expressed as mean ± SD. ** = *p* < 0.01, *** = *p* < 0.001 and **** = *p* < 0.0001

The relevance of H6PD function on susceptibility to anthracycline toxicity was documented by the Kaplan-Meyer analysis of survival. No sham-treated mouse died during the observation period. By contrast, the DXR-induced mortality rate was markedly worsened by the selective impairment of ER/SR-PPP activity. Indeed, 1/14 (7%) and 12/18 (67%) mice died in the WT and H6PD^−/−^ group, respectively (*p* < 0.001, Fig. 2A). Body weight comparably grew during the study period in the untreated mice of both groups (Fig. 2B). This behavior was reverted by DXR treatment that caused a weight loss, whose severity was markedly more pronounced in H6PD^−/−^ mice with respect to WT ones (Fig. 2B).

**Fig. 3:**
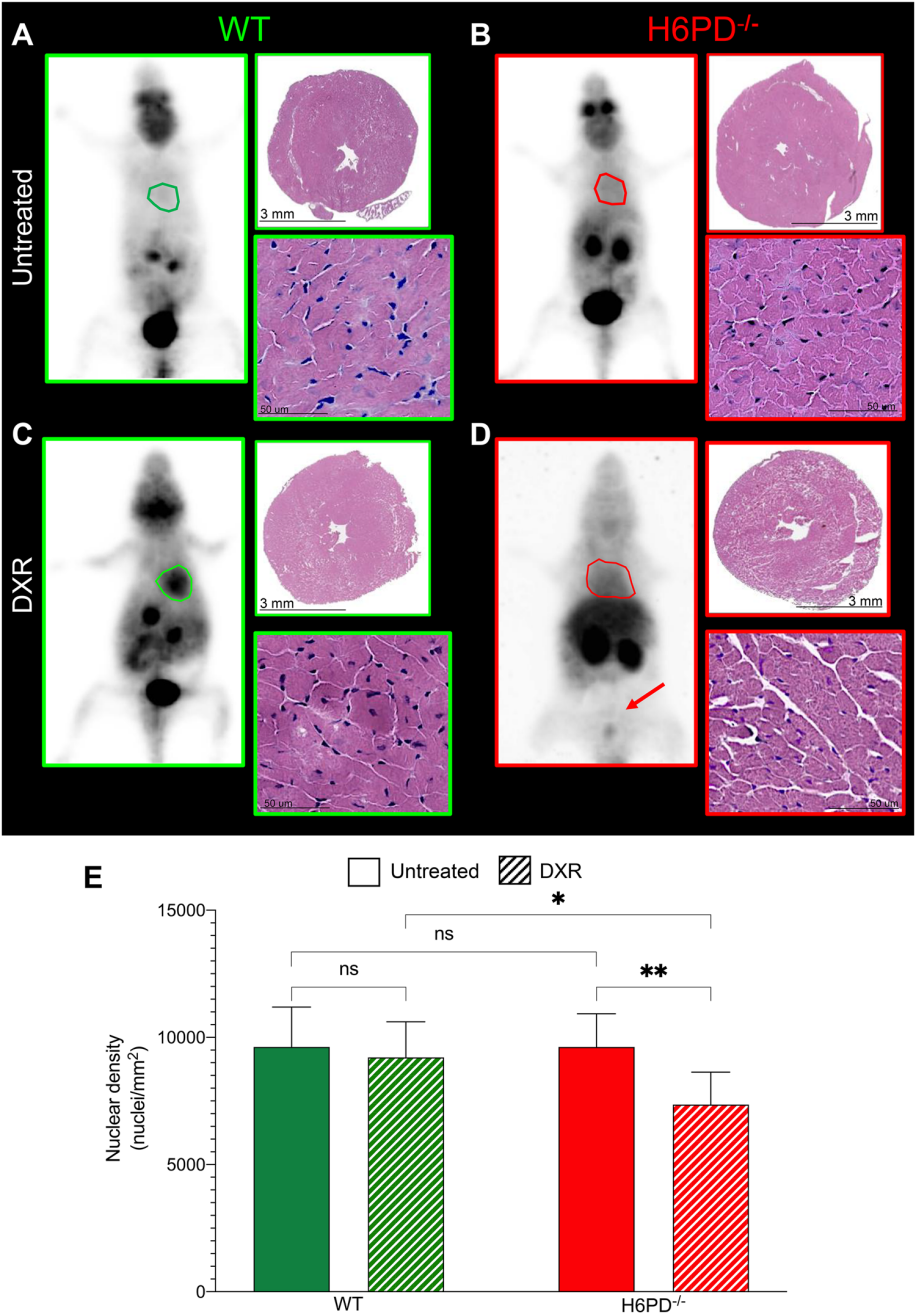
Role of H6PD in FDG retention and myocardial structure under DXR-induced redox stress. Representative images showing FDG average Standardized Uptake Value (SUV) of whole-body mice and hematoxylin–eosin staining of heart harvested from wild-type (WT, panels **A** and **C**) and H6PD knock-out (H6PD^−/−^, panels **B** and **D**) of untreated (panels **A** and **B**) and doxorubicin treated (DXR, panels **C** and **D**) groups. **E** Cardiomyocytes nuclear density evaluated in myocardial section of WT (green columns) and H6PD^−/−^ (red columns) mice of both untreated (solid columns) and DXR (dashed columns) groups. Data are expressed as mean ± SD. * = *p* < 0.05 and ** = *p* < 0.01

PET imaging confirmed that DXR toxicity included cardiac damage. Obviously, PET scans could only be performed in animals surviving the whole study period and thus in 13 WT and 6 KO mice, respectively. Nevertheless, myocardial metabolic response to DXR was largely different in WT and H6PD^−/−^ survivors. While FDG retention was similar in both sham-treated groups (Fig. 2C, D and Fig. 3A, B), DXR treatment increased both indexes of tracer uptake (average and maximal SUV) only in WT models, while it did not affect tracer retention in H6PD^−/−^ mice (Figs. 2C, D and 3C, D). Heart size, analyzed on the same PET images, showed an opposite response since it only—and markedly—increased in DXR-treated H6PD^−/−^ (Fig. 2E, Fig. 3D). Finally, the severity of left ventricular (LV) contractile impairment was indirectly documented by the behavior of bladder volume and radioactivity content that were selectively decreased in DXR-treated H6PD^−/−^ survivors (Fig. 2F, G, Fig. 3D).

### ER/SR-PPP inactivation, DXR-induced cardiac redox stress and mitochondrial damage

At Hematoxylin/Eosin staining, H6PD^−/−^ LV myocardia showed a nuclear density superimposable to the hearts harvested from control mice (Fig. 3A, B and E). Nevertheless, gene deletion enhanced anthracycline cardiotoxicity. Indeed, nuclear density remained unchanged after DXR treatment in WT mice (Fig. 3C and E), while it significantly decreased in the hearts of treated H6PD^−/−^ littermates to values lower with respect to all the other groups (Fig. 3D, E).

**Fig. 4:**
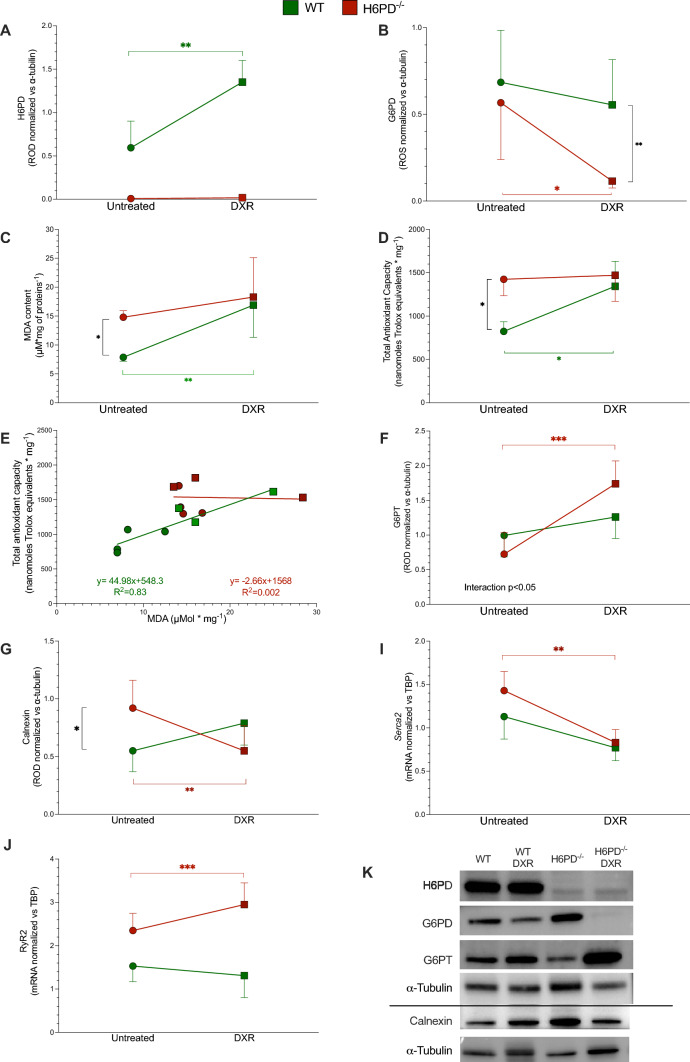
DXR effect on oxidative damage and its impact on Ca^2+^ exchange between ER and mitochondria in H6PD knock-out mice. **A** H6PD and **B** G6PD protein expression, expressed as relative optical density (ROD), in heart homogenates harvested from wild-type (WT, green) and H6PD knock-out (H6PD^−/−^ red) of untreated and doxorubicin treated (DXR) groups. **C** Malondialdehyde concentration, **D** total antioxidant capacity and **E** their correlation in hearts harvested from WT (green) and H6PD^−/−^ (red) of untreated (circles) and DXR (squares) groups. Protein expression of **F** G6PT and calnexin **G** as well as mRNA levels of **H** SERCA2 and **I** RyR2 evaluated in hearts harvested from WT (green) and H6PD^−/−^ (red) mice of untreated (circles) and DXR (squares) groups. **K** Representative western blot signals of H6PD, G6PD G6PT, calnexin proteins and alpha-Tubulin, used as the housekeeping protein **J**. Data are expressed as mean ± SD. * = *p* < 0.05, ** = *p* < 0.01 and *** = *p* < 0.001

WB analysis on tissues harvested soon after sacrifice in surviving animals confirmed that H6PD was not represented in the myocardium of KO mice (Fig. 4A) while its expression was markedly increased by DXR in WT ones. Intriguingly, an almost opposite response was observed when the cytosolic PPP was considered. In KO hearts, G6PD abundance was markedly decreased by DXR that, instead, did not induce any significant change in WT ones (Fig. 4B). The analysis of oxidative damage confirmed the relevance of ER/SR-PPP in the myocardial response to anthracycline treatment, as suggested by the behavior of enzyme abundance. Indeed, MDA content was remarkably higher in H6PD^−/−^ hearts with respect to WT samples before the beginning of therapy (Fig. 4C). Similarly, enzyme deletion was associated with a relative increase in total antioxidant capacity in untreated myocardium of KO mice. DXR selectively increased these indexes in WT mice while being virtually ineffective in H6PD^−/−^ hearts (Fig. 4D). Nevertheless, gene deletion impaired the expected relationship between oxidative damage and total antioxidant capacity. Indeed, Trolox equivalent capacity was directly correlated or independent of MDA content, in WT mice and in H6PD^−/−^ ones, respectively (Fig. 4E).

**Fig. 5:**
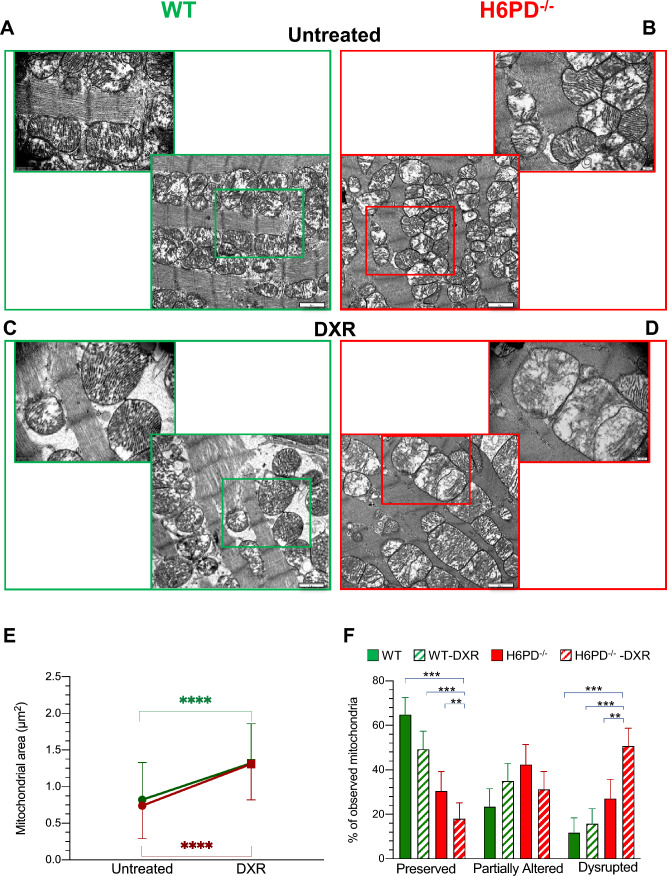
H6PD role in the preservation of mitochondria ultrastructure after DXR-induced redox stress. Representative electron microscopy images of heart harvested from wild-type (WT, panels **A** and **C**) and H6PD knock-out H6PD^−/−^, panels **B** and **D**) of untreated (panels A and B) and doxorubicin treated (DXR, panels C and D) groups. **E** Mitochondrial area of WT (green) and H6PD^−/−^ mice (red) of both untreated and DXR groups. **F** Evaluation of cristae preservation in the myocardium of WT (green columns) and H6PD^−/−^ (red columns) mice of untreated (solid columns) and DXR (red columns) groups. Data are expressed as mean ± SD. ** = *p* < 0.01, *** = *p* < 0.001 and **** = *p* < 0.0001

The relevance of ER/SR-PPP activation in response to DXR-induced redox stress was confirmed by the analysis of the G6P transporter across the reticular membrane: G6PT abundance markedly increased in KO mice, while it remained virtually unaltered in WT ones (Fig. 4F). This response was not related to a possible enlargement of the ER/SR since calnexin levels showed a slight, though not significant, increase in WT mice as opposed to a marked decrease in H6PD^−/−^ myocardium (Fig. 4G and J). Nevertheless, genes encoding key Ca^2+^ handling enzymes were differentially expressed in the hearts from WT vs H6PD-/- mice. Indeed, although DXR decreased *Serca2* levels in both experimental groups, it increased *RyR2* only in H6PD^−/−^ mice (Fig. 4 H, I). The consequent acceleration of SR/ER Ca^2+^ release was followed by a profound mitochondrial damage as documented by TEM (Fig. 5 A–D). Overall, the average area of mitochondria was similar in WT and H6PD^−/−^ hearts, and it comparably increased after anthracycline treatment in untreated WT and H6PD^−/−^ mice, respectively (Fig. 5E). However, the *cristae* number profoundly shrank in treated KO mice as opposed to a slight, and non-significant, decrease in their WT littermates (Fig. 5F).

## Discussion

In the present study, H6PD gene deletion increased by nine-fold the DXR-associated mortality rate without hampering the survival of untreated mice. In agreement with the recognized pro-oxidant activity of DXR, the data document the redox imbalance as a main molecular phenotype underlying DXR-elicited excess mortality secondary to H6PD deletion. By contrast, the endurance of WT hearts to the DXR-related redox stress matched an enhanced H6PD expression, as opposed to the behavior of G6PD, whose levels remained unchanged or even decreased after chemotherapy. Thus, the antioxidant defense against the DXR-induced oxidative damage is critically dependent on the activation of ER/SR-PPP rather than its cytosolic counterpart.

As e second point, the present data extend to the myocardium the mandatory role of ER/SR-PPP in modulating FDG uptake. The missing response of tracer retention to DXR in mice devoid of H6PD catalytic function matched the analysis of both molecular phenotype and structural damage. Thus, in agreement with the notion that cardiac injury is a main feature of anthracyclines toxicity, these data provide a mechanistic explanation of the previously reported predictive power of cardiac FDG uptake in cancer patients.

### Mandatory role of ER/SR PPP in the cardiac response to DXR-induced redox stress

Most recognized antioxidant pathways are immediately activated by redox stress and are dependent upon the NADPH reductive power. The regeneration of this cofactor is thought to be a largely cytosolic process involving the oxidative arm of the G6PD-triggered PPP with a relatively minor contribution of other pathways [26]. Indeed, G6PD deficiency aggravates contractile impairment in experimental models of oxidative damage caused by myocardial infarction, ischemia–reperfusion injury, or pressure overload [27]. However, the response to DXR-induced redox stress seems to follow a different sequence of events: it is not immediate, being activated up to 4 weeks after the occurrence of the oxidative damage [28] and, more importantly, it is largely independent of G6PD catalytic function [5]. These features suggest that the DXR-induced oxidative damage might be confined to cell compartments surrounded by membranes impermeable to the reductive power conveyed by polar cofactors.

The synthetic lethality of H6PD gene deletion and DXR treatment confirms this hypothesis. Indeed, it indicates that the high ER/SR vulnerability to anthracyclines [3, 4] is at least partially counterbalanced by a locally confined pathway—the H6PD-triggered PPP—able to empower the same ER/SR with a local and autonomous antioxidant capability. This myocardial response to the DXR-related redox stress seems to reflect the activation of an integrated mechanism able to simultaneously accelerate the G6P access to the reticular lumen and the rate of its dehydrogenation. Indeed, besides the increase in H6PD level in WT mice, DXR also accelerated G6PT expression in both groups. G6PT is the carrier dedicated to the G6P transport through the reticular membrane impermeable to this polar metabolite [29]. Its increase was obviously ineffective in KO mice. Nevertheless, this response suggests per se the presence of a local sensor able to adapt G6P access to H6PD catalytic function as a regulator of the ER/SR redox status.

Accordingly, these data indicate that cardiomyocytes’ response to the DXR-induced oxidative damage implies a coordinated acceleration of G6P flux through the ER/SR-PPP. By contrast, this same response seems not to involve its most recognized cytosolic counterpart, as DXR did not substantially affect (in WT hearts) or even decreased (in KO ones) G6PD levels.

### Reticular PPP, Ca^2+^ exchanges and mitochondrial function

In the preliminary phase, we verified the H6PD docking to the reticular membrane and the ER/SR loss of glucose or G6P dehydrogenation capability in the studied H6PD^−/−^ model. This evaluation was limited to hepatocytes to overcome the technical limitations in harvesting adequate ER samples from the myocardium, and to facilitate the comparison with previous literature [20]. Microsomes of WT mice were the unique cell fraction able to dehydrogenate glucose in the presence of NADP. Since this reaction cannot be catalyzed by G6PD, this finding confirmed the expected H6PD binding to the WT reticular membranes. The same WT microsomes also showed a high capability to dehydrogenate G6P, possibly suggesting a coexistent ER/SR localization of G6PD. However, this explanation seems unlikely according to two considerations. On one side, the rate of G6P conversion to 6P-gluconate agreed with the previously quoted high capability of H6PD in accelerating this reaction [20]. On the other side, this same catalytic function was absent in the microsomes of KO mice. Accordingly, this preliminary characterization documented the selective H6PD binding to the reticular membranes and the absence of G6PD in this compartment.

From the ultrastructural point of view, TEM analysis of hepatocytes confirmed the previously documented pivotal role of reticular PPP in preserving the ER/SR-mitochondria networking [30]. Although not directly evaluated, several observations extend this concept to the myocardium. On one side, DXR almost halved calnexin levels in H6PD^−/−^ mice, as opposed to the increase that was observed in WT ones in agreement with previous studies [31]. More importantly, combining gene deletion and anthracycline therapy markedly impaired the reticular accumulation of Ca^2+^, since it simultaneously slowed-down its input (by decreasing *Serca2* expression) [32] and accelerated its output (by upregulating *RyR2* expression) [33].

The impairment in reticular Ca^2+^ accumulation expectedly resulted in a severe mitochondrial impairment [30, 31]. Indeed, the myocardium of treated H6PD^−/−^ mice showed an evident mitochondrial damage as documented by the TEM evidence of swelling and *cristae* disruption [34]. In agreement with previous evidence in cancer [35], these features suggest the opening of the mitochondrial permeability transition pore [36] that represents an acknowledged trigger of pathways underlying DXR-induced cardiomyocyte necrosis [37]. This hypothesis is confirmed by the evidence that myocardial nuclear density was similar in all samples but those harvested from treated H6PD^−/−^ mice, in which this sign of cardiomyocytes’ loss reproduced the severity observed in models of overt DXR-induced cardiac failure [38].

### Functional evidence of ER/SR-PPP role in myocardial response to DXR toxicity

Besides the evaluation of its molecular determinants, the ER/SR-PPP activation in response to DXR was directly documented by functional imaging with PET. In agreement with previous studies [8], the response of WT hearts to chemotherapy combined increased H6PD levels and enhanced FDG uptake values. By contrast, tracer retention remained unchanged in H6PD^−/−^ ones.

According to classical models [39], this divergence would be interpreted as a sign of different glucose consumption rates in the hearts of WT and KO mice. Nevertheless, this interpretation has been challenged by a series of evidence [14] and, mostly, by the observation that FDG uptake and glucose consumption show an opposite response to DXR in cultured H9c2 myoblasts [8]. This disagreement was not directly verified in our in vivo models. Nevertheless, the absent response of KO hearts to the same drug corroborates the mandatory role of H6PD function in FDG uptake and provides a mechanistic explanation for the prognostic penetrance of myocardial tracer retention in DXR-treated patients [13].

### Limitations

Several limitations need to be considered for a correct interpretation of the present data. As a first point, the cumulative dose (24 mg/Kg), selected according to previous experience in experiments dedicated to DXR cardiotoxicity [40], was almost two-fold higher than the clinical one [41] and might thus have contributed to the extremely high mortality rate observed in H6PD^−/−^ mice. Accordingly, although early and late toxicity share similar underlying mechanisms [28], further studies should be performed to define the role of ER/SR-PPP activation in the progressive development of heart failure in the clinical setting.

Dynamic PET acquisition permitted us to adequately analyze urinary output without the interference of possible voiding occurring after tracer injection and before late scan. Despite the availability of the complete acquisition, the kinetics of FDG accumulation in the myocardium was not analyzed. Indeed, the spillover related to the limited spatial resolution of microPET detection would have differentially affected the measurements in models with preserved or enlarged LV volume. Nevertheless, the analysis of late myocardial tracer concentration confirmed the previous clinical observation reporting an increased cardiac avidity for FDG in anthracycline-treated patients and experimental mice [8, 13].

LV function was not directly evaluated, while the hemodynamic impairment was only indirectly identified by the simultaneous observation of LV dilation and decreased urinary output. Accordingly, the present data do not directly document the occurrence of an early dysfunction after the administered high DXR doses. Nevertheless, this limitation does not compromise the main finding of the present study, *i.e.*, the mandatory role of the ER/PPP in the myocardial defense mechanisms against the anthracycline-induced redox stress.

## Conclusions

In the present study, switching off the ER/SR-PPP markedly aggravated DXR cardiotoxicity. This life-threatening effect was associated with a profound redox imbalance coupled with alterations of mitochondrial structure and a significant loss of cardiomyocytes. To the best of our knowledge, these findings represent the first evidence documenting that the recognized vulnerability of ER/SR to the anthracycline oxidative damage is counterbalanced by a compartmentalized pathway able to restore the redox equilibrium within the reticular lumen.

The present data do not elucidate whether and how the G6P processing machine confined within the ER/SR interacts with the most recognized cytosolic G6PD-triggered pathway. Nevertheless, the evident interplay between redox control and Ca^2+^ exchanges between the ER/SR and the mitochondria of cardiomyocytes, shape the reticular PPP as a potential new target for strategies aimed to decrease anthracycline toxicity. Similarly, these same data also indicate that the control of redox stress and its triggering mechanisms might be not shared by all cellular compartments, at least in cardiomyocytes. In other words, the analysis of ROS generation and its consequences should take into account the possibility of a heterogeneous distribution of the oxidative stress and antioxidant defenses in the different cellular compartments.

On the clinical ground, the strict link between FDG uptake and its primary response to DXR potentially improves the informative content of routine PET/CT imaging since the early diagnosis of cardiotoxicity would permit to anticipate therapies for cardiovascular risk prevention in cancer patients [41]. On a translational point of view, the noninvasive nature of this tool might facilitate the development of experimental strategies aimed to counteract anthracycline cardiotoxicity. Finally, from a basic science perspective, these data configure myocardial FDG retention as an index of ER/SR-PPP activation, rather than a pure marker of glycolytic flux. This new interpretative model might profoundly modify the current interpretation of cardiac FDG uptake, potentially opening new windows on the myocardial antioxidant responses to different stressors.

## Data Availability

Enquiries about data availability should be directed to the authors.
